# Supplementary material for: A Critical View of the Application of the APEX Software (Aqueous Photochemistry of Environmentally-Occurring Xenobiotics) to Predict Photoreaction Kinetics in Surface Freshwaters
Source: Molecules. 2019 Dec 18;25(1):9. doi: 10.3390/molecules25010009 (PMC7017383; doi:10.3390/molecules25010009)
Supplement: Supplementary file 1 [file molecules-25-00009-s001.zip › 3.2.4_gcc-4.4.0.zip!/3.2.4_gcc-4.4.0/3.2.4_gcc-4.4.0/doc/octave/html/interpreter/Multiple-Return-Values.html]

Multiple Return Values - Untitled


Next: Variable-length Argument Lists,
Previous: Defining Functions,
Up: Functions and Scripts


---

### 11.2 Multiple Return Values

Unlike many other computer languages, Octave allows you to define
functions that return more than one value. The syntax for defining
functions that return multiple values is

```
     function [ret-list] = name (arg-list)
       body
     endfunction
```

where name, arg-list, and body have the same meaning
as before, and ret-list is a comma-separated list of variable
names that will hold the values returned from the function. The list of
return values must have at least one element. If ret-list has
only one element, this form of the `function` statement is
equivalent to the form described in the previous section.

Here is an example of a function that returns two values, the maximum
element of a vector and the index of its first occurrence in the vector.

```
     function [max, idx] = vmax (v)
       idx = 1;
       max = v (idx);
       for i = 2:length (v)
         if (v (i) > max)
           max = v (i);
           idx = i;
         endif
       endfor
     endfunction
```

In this particular case, the two values could have been returned as
elements of a single array, but that is not always possible or
convenient. The values to be returned may not have compatible
dimensions, and it is often desirable to give the individual return
values distinct names.

In addition to setting `nargin` each time a function is called,
Octave also automatically initializes `nargout` to the number of
values that are expected to be returned. This allows you to write
functions that behave differently depending on the number of values that
the user of the function has requested. The implicit assignment to the
built-in variable `ans` does not figure in the count of output
arguments, so the value of `nargout` may be zero.

The `svd` and `lu` functions are examples of built-in
functions that behave differently depending on the value of
`nargout`.

It is possible to write functions that only set some return values. For
example, calling the function

```
     function [x, y, z] = f ()
       x = 1;
       z = 2;
     endfunction
```

as

```
     [a, b, c] = f ()
```

produces:

```
     a = 1
     
     b = [](0x0)
     
     c = 2
```

along with a warning.

— Built-in Function: **nargout** ()  
— Built-in Function: **nargout** (fcn\_name)  
> Within a function, return the number of values the caller expects to
> receive. If called with the optional argument fcn\_name, return the
> maximum number of values the named function can produce, or -1 if the
> function can produce a variable number of values.
>
> For example,
>
> ```
>           f ()
> ```
>
> will cause `nargout` to return 0 inside the function `f` and
>
> ```
>           [s, t] = f ()
> ```
>
> will cause `nargout` to return 2 inside the function
> `f`.
>
> At the top level, `nargout` is undefined.
>
>
>
> **See also:** nargin, varargin, varargout.

— Function File: msgstr = **nargchk** (minargs, maxargs, nargs)  
— Function File: msgstr = **nargchk** (minargs, maxargs, nargs, "string")  
— Function File: msgstruct = **nargchk** (minargs, maxargs, nargs, "struct")  
> Return an appropriate error message string (or structure) if the
> number of inputs requested is invalid.
>
> This is useful for checking to see that the number of input arguments
> supplied to a function is within an acceptable range.
>
>
>
> **See also:** nargoutchk, error, nargin, nargout.

— Function File: msgstr = **nargoutchk** (minargs, maxargs, nargs)  
— Function File: msgstr = **nargoutchk** (minargs, maxargs, nargs, "string")  
— Function File: msgstruct = **nargoutchk** (minargs, maxargs, nargs, "struct")  
> Return an appropriate error message string (or structure) if the
> number of outputs requested is invalid.
>
> This is useful for checking to see that the number of output
> arguments supplied to a function is within an acceptable range.
>
>
>
> **See also:** nargchk, error, nargout, nargin.
